# Real-time quantitative reverse transcription polymerase chain reaction detection of SARS-CoV-2 Delta variant in Canadian wastewater

**DOI:** 10.14745/ccdr.v49i05a07

**Published:** 2023-05-01

**Authors:** Shelley Peterson, Jade Daigle, Codey Dueck, Audra Nagasawa, Michael Mulvey, Chand S Mangat

**Affiliations:** 1 Wastewater Surveillance Unit, National Microbiology Laboratory, Public Health Agency of Canada, Winnipeg, MB; 2 Centre for Population Health Data, Statistics Canada, Ottawa, ON; 3 Department of Medical Microbiology and Infectious Diseases, Max Rady College of Medicine, University of Manitoba, Winnipeg, MB

**Keywords:** SARS-CoV-2, wastewater, variant, Delta, B.1.617.2, qPCR

## Abstract

**Background:**

Severe acute respiratory syndrome coronavirus 2 (SARS-CoV-2) variants of concern are associated with increased infectivity, severity, and mortality of coronavirus disease 2019 (COVID-19) and have been increasingly detected in clinical and wastewater surveillance in Canada and internationally. In this study, we present a real-time quantitative reverse transcription polymerase chain reaction (RT-qPCR) assay for detection of the N gene D377Y mutation associated with the SARS-CoV-2 Delta variant in wastewater.

**Methods:**

Wastewater samples (n=980) were collected from six cities and 17 rural communities across Canada from July to November 2021 and screened for the D377Y mutation.

**Results:**

The Delta variant was detected in all major Canadian cities and northern remote regions, and half of the southern rural communities. The sensitivity and specificity of this assay were sufficient for detection and quantitation of the Delta variant in wastewater to aid in rapid population-level screening and surveillance.

**Conclusion:**

This study demonstrates a novel cost-effective RT-qPCR assay for tracking the spread of the SARS-CoV-2 Delta variant. This rapid assay can be easily integrated into current wastewater surveillance programs to aid in population-level variant tracking.

## Introduction

The severe acute respiratory syndrome coronavirus 2 (SARS-CoV-2) pandemic began in Wuhan, China in late 2019 before becoming a worldwide pandemic in 2020. Beginning in September 2020, variants of concern (VOC) began to emerge which had mutations leading to increased viral transmission rates, increased virulence, or the ability to escape existing vaccines ((1–4)). On May 11, 2021, the World Health Organization declared the Delta variant (B.1.617.2) to be a VOC ((5)). The Delta variant has been shown to be both more transmissible and more virulent than the wild-type (WT) (Wuhan) strain ((6–8)).

Wastewater-based epidemiology (WBE) has proven to be a powerful tool for tracking the spread of SARS-CoV-2 on a population level, and recently has become instrumental in monitoring the dissemination of VOC across Canada and throughout the world ((9–12)). The real-time quantitative reverse transcription polymerase chain reaction (RT-qPCR)-based assays have previously been developed to identify mutations associated with emerging VOC including Alpha (Sdel69-70, ND3L), Beta (Sdel241, N501Y) and Gamma (N501Y) ((13–15)). Early detection of VOC can potentially lead to improved public health responses, such as increased sequencing of clinical isolates, enhanced surveillance and enhanced public health measures. Monitoring relative amounts of SARS-CoV-2 variants over time can be useful for monitoring trends in viral transmission and potentially assessing the effectiveness of public health interventions ((16)).

In this report, we describe a novel RT-qPCR assay for detection of the N gene D377Y allele—associated with the Delta variant—in wastewater. We applied this assay to wastewater samples collected from 36 sampling locations drawn from 23 Canadian cities and towns, both remote and urban, to monitor the dissemination of the Delta variant throughout the country. This population-level surveillance approach could be instrumental for monitoring changes in VOC prevalence and effects of public health interventions for reduction of viral spread within health regions.

## Methods

### Sample collection and nucleic acid extraction

Wastewater was collected between June 30 and December 1, 2021, from 16 urban wastewater treatment plants (WWTP) from six cities along with 20 WWTP and lift stations from 17 towns and rural locations in Canada. Fifteen of the WWTP from five cities were sampled as part of Statistics Canada’s Canadian Wastewater Survey ((17)). A 24-hour composite sample was collected three times per week at each treatment facility and shipped to the National Microbiology Laboratory at 4°C. The samples were stored at 4°C for up to 24 hours until processed.

A 300 ml sample of primary post-grit influent or raw wastewater was mixed by inversion, then a 30 mL aliquot was drawn and processed as previously described ((14)). RNA was extracted using the MagNA Pure 96 DNA and Viral NA Large Volume Kit (Roche Diagnostics, Laval, Québec) using the Plasma External Lysis 4.0 protocol as per manufacturer instructions.

### Delta variant of concern assay design

An assay was developed to detect the D377Y mutation consisting of a G->T in the N gene (G29406T) due to relative rarity in the general Canadian population of SARS-CoV-2 genomes, and relative exclusivity within the Delta variant genome (*personal communication, G. Van Domselaar*). This assay was designed to detect both WT and variant (V) sequences for each allele, allowing for discrimination between V and WT SARS-CoV-2 RNA.

The WT SARS-CoV-2 (NC_045512.1) sequence, along with Delta variant sequences (EPI_ISL_1372093, EPI_ISL_2134533, EPI_ISL_2134644, EPI_ISL_2134933, EPI_ISL_2135087), were obtained from Global Initiative on Sharing Avian Influenza Data ((18)) and used for primer and probe design. Oligonucleotide primers and probes were chosen for each target region using Primer Express Software v3.0 (Thermo Fisher Scientific, Waltham, Massachusetts) and Primer3 v4.1.0 ((19)). Linear dsDNA oligonucleotide gene fragments (Integrated DNA, Coralville, Iowa) consisting of the gene region flanking the variant region for either the WT or variant sequence (**Table A1**) were employed as standards and quantified using a One-Step RT-ddPCR Advanced Kit for Probes (Bio-Rad, Mississauga, Ontario) on a QX200 Droplet Digital PCR System (Bio-Rad).

Real-time quantitative reverse transcription polymerase chain reaction assay conditions

RT-qPCR was performed for D377Y WT and V assays, along with the United States Centers for Disease Control and Prevention N1 and N2 assays and interpreted as previously described ((14,20)) with concentrations of 500 nM of each primer (D377Y_F: CATTCCCACCAACAGAGCCT, D377Y_R: TGTCTCTGCGGTAAGGCTTG) and 500 nM of each probe (D377Y_WT: AGAAGGCTGATGAAA, D377Y_V: AGAAGGCTTATGAAAC). Each real-time PCR was performed in duplicate or triplicate as indicated with the appropriate non-template controls and positive controls.

### Determination of limit of detection

The assay limit of detection (LOD) was assessed as the lowest concentration at which there was >95% test positivity in 15 replicates of a 1.5-fold serial dilution series from 45 copies/reaction (cp/rxn) to 1.8 cp/rxn of dsDNA oligonucleotide standards.

### Data analyses

Amplification efficiencies (E) were calculated using E=−1 + 10^(-1/slope)^ x 100. Data analyses were performed using R version 4.1.1 on Rstudio using the tidyverse packages ((21)).

## Results

The assay limits of detection were 4 cp/rxn (WT) and 3 cp/rxn (V) when measured as a pure specimen without interfering alleles. These LODs were near the theoretical limit of RT-qPCR and sufficient for sensitive detection in wastewater, where SARS-CoV-2 RNA concentrations can be very low. Standard curves were as follows: WT (slope=−3.45, intercept=38.52, R^2^=0.999); and V (slope=−3.29, intercept=38.25, R^2^=0.999). The amplification efficiencies of the WT and V reactions were 101% and 95%, respectively.

**Figure 1:**
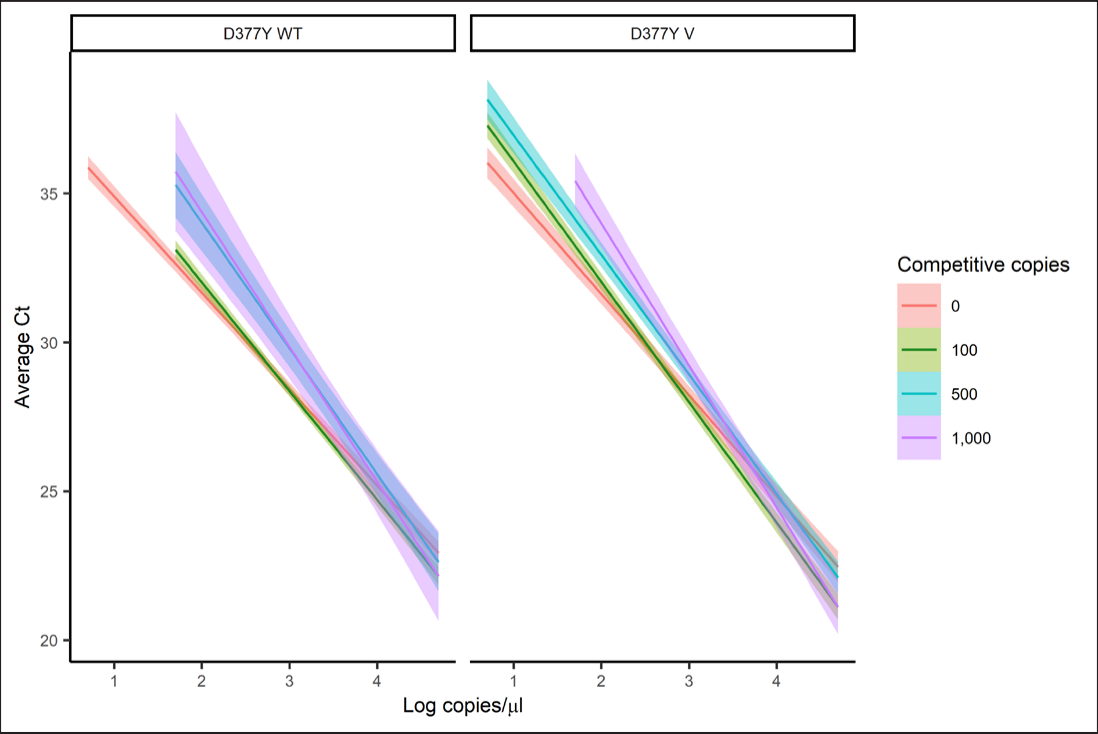
Standard curves for D377Y assays in the presence of the alternate genotype for each allele^a^ Abbreviations: Ct, cycle threshold; SARS-CoV-2, severe acute respiratory syndrome coronavirus 2; V, variant; WT, wild-type ^a^ Standard curves for real-time quantitative reverse transcription polymerase chain reaction SARS-CoV-2 wild-type and D377Y variant B.1.617 assays against tenfold dilutions of DNA oligonucleotide controls in the presence of 100, 500 and 1,000 copies/µL of the alternate genotype for each allele

As relative amounts of WT and V template within wastewater samples may vary considerably, standard curves were also created for each assay in the presence of 100, 500 and 1,000 cp/µL of the alternate allele to assess their stability (**Figure 1**). Presence of the WT template had a limited effect on V template detection with the variant assay, with a loss of signal at 1 cp/µL V only in the presence of 1,000 cp/µL WT; a concentration much higher than is likely to be detected in wastewater. Presence of V template led to decreased sensitivity and increased range of error of the WT assay, with detection at 10 cp/µL WT, but not 1 cp/µL WT in the presence of any concentration of V template. Standard deviations of both assays were determined using the alternate alleles in this experiment to assess variance over a range of concentrations. Standard deviations were averaged across three concentrations (100 cp/µL, 500 cp/µL and 1,000 cp/µL) and were 0.38 Ct (WT) and 0.31 (V) compared with 0.09–0.18 for previously published assays ((14)).

**Figure 2:**
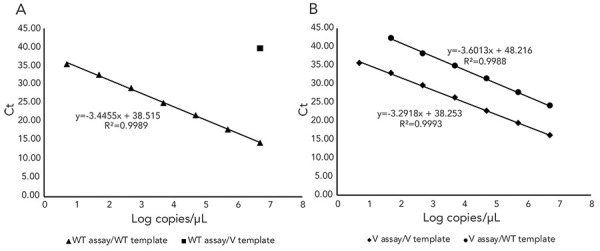
Standard curves^a,b,c^ for the D377Y real-time polymerase chain reaction assays performed with serial dilutions of synthetic DNA oligonucleotides for the wild-type and D377Y variant alleles Abbreviations: Ct, cycle threshold; V, variant; WT, wild-type ^a^ For all standard curves, equations for the lines and R^2^ values are indicated ^b^ (A) Standard curve for the WT assay using WT template and cross-reactivity with the V template ^c^ (B) Standard curve for the V assay using V template (solid line) and cross-reactivity with the WT template (dashed line)

To test analytical specificity, both WT and V targets were tested in triplicate against serial dilutions from 10^6^ cp/µL to 10^0^ cp/µL of the alternate allele oligonucleotide. The WT assay showed negligible cross-reactivity, with a 25 Ct delayed detection in the presence of 1 x 10^6^ cp/µL WT. The V assay showed cross reactivity with the WT template; however, the amplification was delayed by ~8 Ct (**Figure 2**).

**Figure 3:**
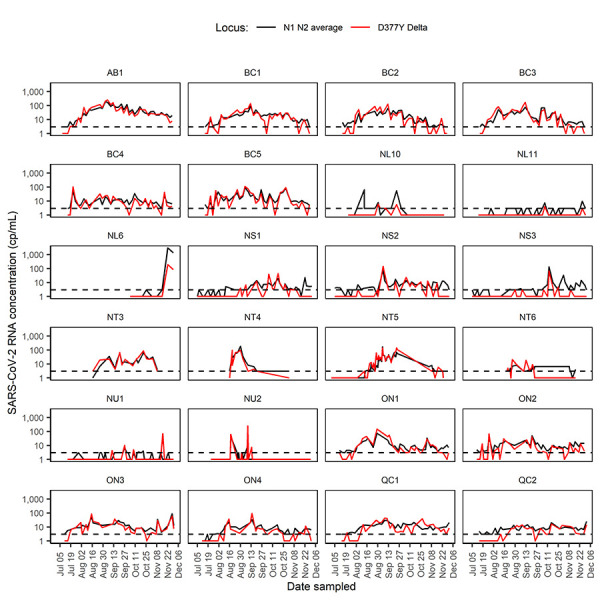
Detection of SARS-CoV-2 Delta variant in wastewater from Canadian cities and rural areas using real-time quantitative reverse transcription polymerase chain reaction^a,b^ Abbreviations: AB, Alberta; BC, British Columbia; NL, Newfoundland and Labrador; NS, Nova Scotia; NT, Northwest Territories; NU, Nunavut; ON, Ontario; QC, Québec; RNA, ribonucleic acid; SARS-CoV-2, severe acute respiratory syndrome coronavirus 2 ^a^ Red line depicts copies/ml (cp/mL) of D377Y mutation indicative of Delta variant presence ^b^ Black line is the SARS-CoV-2 concentration using the average of the Centers for Disease Control and Prevention N1 and N2 cp/mL. Sites tested over a period of less than one month or with fewer than three samples in which SARS-CoV-2 was detected by N1 or N2 are not shown. Dashed line represents the limit of quantification of the assay. Sites are described in Table A2

A total of 980 samples from 36 urban and remote WWTPs and lift stations across Canada were sampled from June 30 to December 1, 2021 (**Table A2**). Of these, 539 (55%) tested positive for the SARS-CoV-2 Delta variant D377Y mutation, 210 (21.4%) tested positive for N1/N2 only and 232 (23.6%) samples were negative for both N1/N2 and D377Y. Additionally, there were 8 (0.8%) samples in which SARS-CoV-2 was detected by the D377Y assays but not N1/N2, of which seven had D377Y detection in only one of two replicates and six had detection <10 cp/mL. The Delta variant was detected in all six major cities, with initial detection ranging from July 11 to August 30 in the larger cities and October 7 in NL11 (St. John’s) (**Figure 3**). Delta variant signal was initially detected in the majority of cities between July 17 and 22. The peak signal (highest concentration of Delta detected) in the cities throughout the study period ranged from July 22 to October 18, averaging 32 days after initial detection (range: 0–66, IQR: 10–49). Following initial detection in five of the six cities, Delta signal rapidly increased, becoming roughly equivalent to the SARS-CoV-2 N1 + N2 signal throughout the remainder of the study period. This sharp increase is indicative of rapid displacement of other circulating variants by Delta, as seen in clinical cases by genomic surveillance of SARS-CoV-2 variants. In St. John’s, Newfoundland and Labrador, Delta signal was detected only twice during the study period and SARS-CoV-2 signal remained low; a pattern typically seen in the more remote locations in this study.

The more sparsely populated regions investigated in this study showed less consistent detection of D377Y, with detection in 50% of remote sites in Newfoundland and Labrador (n=6/12) during at least one time point throughout the sampling period. In Newfoundland and Labrador, D377Y was not detected in 4/6 sites prior to the last week of October 2021, whereas the remaining two sites had detection in July and September.

In the northern Territorial regions, Delta signal was detected in all six Northwest Territories (NWT) sites and both Nunavut (NU) sites during at least one time point. The NU1 variant had sporadic low-level detection in September, while D377Y was detected in NU2 in August to mid-September, peaking with a strong signal on September 8. The NT1 variant was sampled only in November, with high levels of detection throughout the month. Delta signal was first detected between August 12–19 in four of the remaining five NWT sites, and on September 20 for the final site. Four NWT sites (NT1,NT4–NT6) were not sampled during the month of October.

## Discussion

This study describes the development of RT-qPCR assays to detect the N gene D377Y mutation associated with the SARS-CoV-2 Delta variant. The LODs of this assay were near the theoretical limit of RT-qPCR and sufficient for sensitive detection in wastewater, where SARS-CoV-2 RNA concentrations can be very low. The robustness and sensitivity of the V component of the assay allows for trending analysis, and where appropriate, early warning detection in communities and for monitoring the decline of the Delta wave.

The D377Y V assay is valuable for tracking the spread of the Delta variant in wastewater as it was used to monitor the spread of the Delta variant in eight major cities and 26 towns and rural locations across Canada over a four-month period. The Delta variant was detected in wastewater from all major Canadian cities, with a rapid increase in signal shortly following onset of detection, indicating rapid spread of Delta and displacement of other variants. Delta signal was also observed in approximately half of rural locations in Southern Canada, and all locations in Northern Canada. These data demonstrate the utility of this assay for tracking the spread of the SARS-CoV-2 Delta variant. These one-step RT-qPCR assays can be easily integrated into currently used wastewater surveillance programs to aid in SARS-CoV-2 surveillance.

## Limitations

Assay limitations include the loss in sensitivity in the presence of the V allele, which limits the interpretation of the WT component of the assay during the onset of a Delta wave, where high levels of variant genomic material will attenuate the WT signal. This is consistent with previous studies that found a similar level of cross-reactivity between variant and WT assays ((13,22)). In wastewater samples, this delayed cross-reactivity is negligible, as the SARS-CoV-2 concentration is very low. Other limitations of this assay include: 1) inconsistent detection when RNA concentration in samples approaches the LOD of the assay or 2) the presence of inhibitors found in wastewater. Limitations of wastewater-based surveillance include testing being limited to populations present within the wastewater catchment area, variations in viral shedding between SARS-CoV-2 variants and infected individuals, and variations in wastewater composition due to weather or industrial events.

The Delta variant of SARS-CoV-2 is defined by 27 mutations, which are commonly detected by whole genome sequencing (3,23–25)). While detection of one mutation such as D377Y is not determinative of Delta variant presence, it is highly indicative as the N gene D377Y mutation is found very rarely in non-Delta strains ((26)).

## Conclusion

Surveillance using RT-qPCR is a rapid and cost-effective method of screening for SARS-CoV-2 variants in both wastewater and clinical specimens. These assays provide a complement to SARS-CoV-2 variant detection assays as previously described ((14)) for surveillance of SARS-CoV-2 variants in wastewater. Wastewater-based surveillance is a valuable tool for tracking the spread of SARS-CoV-2 variants on a population level in regions where clinical testing is limited. The relative fraction of the Delta variant measured in wastewater using the assay developed in this work was communicated to public health decision-makers by weekly reporting across a network of surveillance sites throughout Canada. To our knowledge, these data were used as a complimentary public health intelligence stream and not directly actioned. Thus, over the course of the pandemic, this was principally the use for wastewater surveillance data amongst infectious control and public health leadership; likely because of a gap in trust arising from a lack of precedent and unfamiliarity with the data, in addition to the public scrutiny and pressure associated with pandemic. We hope that this work and the work of others will establish a base of use cases that will improve the action-ability of wastewater surveillance. A conservative use case could be to maintain wide-scale infectious control measures based on wastewater surveillance data. While more than a year has passed since the Delta wave, the relevance of the assay described here remains as sub-lineages of the Delta VOC has been observed in wild populations of white-tailed deer ((27,28)) and this work could contribute to the monitoring of the expanding host range of this virus. With the high number of asymptomatic COVID-19 cases and limited testing capacity worldwide, augmentation of surveillance capabilities by monitoring spread of SARS-CoV-2 variants in wastewater can aid in public health efforts.

## Appendix

**Table A1:** Positive control gBlock sequences for the SARS-CoV-2 Delta variant real-time quantitative reverse transcription polymerase chain reaction assays

Region	Allele	Sequence
N D377Y	WT	AAAGATCCAAATTTCAAAGATCAAGTCATTTTGCTGAATAAGCATATTGACGCATACAAAACATTCCCACCAACAGAGCCTAAAAAGGACAAAAAGAAGAAGGCTGATGAAACTCAAGCCTTACCGCAGAGACAGAAGAAACAGCAAACTGTGACTCTTCTTCCTGCTGCAGATTTGGATGATTTCTCCAAACAATTGCAA
	Variant	AAAGATCCAAATTTCAAAGATCAAGTCATTTTGCTGAATAAGCATATTGACGCATACAAAACATTCCCACCAACAGAGCCTAAAAAGGACAAAAAGAAGAAGGCTTATGAAACTCAAGCCTTACCGCAGAGACAGAAGAAACAGCAAACTGTGACTCTTCTTCCTGCTGCAGATTTGGATGATTTCTCCAAACAATTGCAA

Abbreviations: SARS-CoV-2, severe acute respiratory syndrome coronavirus 2; WT, wild-type

**Table A2:** Location and number of samples tested from wastewater treatment plants and lift stations across Canada

Site code	Region	Sampling date range	Date of first detection	Date of peak signal	Number of samples
AB1	Edmonton, AB	2021-07-08 to 2021-11-28	2021-07-18	2021-09-05	40
BC1	Vancouver, BC	2021-07-15 to 2021-11-28	2021-07-22	2021-09-12	37
BC2	Vancouver, BC	2021-07-15 to 2021-11-28	2021-07-22	2021-09-12	37
BC3	Vancouver, BC	2021-07-15 to 2021-11-28	2021-07-22	2021-09-12	38
BC4	Vancouver, BC	2021-07-15 to 2021-11-28	2021-07-22	2021-07-22	37
BC5	Vancouver, BC	2021-07-15 to 2021-11-28	2021-07-22	2021-09-05	38
NL1	Newfoundland and Labrador	2021-10-04 to 2021-11-29	Not detected	Not detected	9
NL2	Newfoundland and Labrador	2021-10-04 to 2021-11-29	2021-11-29	2021-11-29	9
NL3	Newfoundland and Labrador	2021-10-04 to 2021-11-29	2021-11-16	2021-11-16	8
NL4	Newfoundland and Labrador	2021-07-19 to 2021-11-24	Not detected	Not detected	14
NL5	Newfoundland and Labrador	2021-10-06 to 2021-11-17	Not detected	Not detected	6
NL6	Newfoundland and Labrador	2021-10-04 to 2021-11-29	2021-11-22	2021-11-22	9
NL7	Newfoundland and Labrador	2021-07-14 to 2021-11-24	2021-07-21	2021-07-21	20
NL8	Newfoundland and Labrador	2021-09-16 to 2021-11-29	Not detected	Not detected	8
NL9	Newfoundland and Labrador	2021-10-14 to 2021-11-30	Not detected	Not detected	7
NL10	Newfoundland and Labrador	2021-07-22 to 2021-11-24	2021-08-30	2021-08-30	20
NL11	Newfoundland and Labrador	2021-07-13 to 2021-11-29	2021-09-02	2021-10-07	39
NL12	Newfoundland and Labrador	2021-10-04 to 2021-11-30	2021-10-25	2021-10-25	9
NL13	Newfoundland and Labrador	2021-10-14 to 2021-11-30	Not detected	Not detected	7
NS1	Halifax, Nova Scotia	2021-07-05 to 2021-12-01	2021-08-23	2021-10-18	42
NS2	Halifax, Nova Scotia	2021-07-05 to 2021-12-01	2021-08-04	2021-09-06	42
NS3	Halifax, Nova Scotia	2021-07-05 to 2021-12-01	2021-08-30	2021-10-13	42
NT1	Northwest Territories	2021-11-03 to 2021-11-24	2021-11-04	2021-11-10	15
NT2	Northwest Territories	2021-10-19 to 2021-11-17	2021-09-20	2021-09-20	11
NT3	Northwest Territories	2021-08-16 to 2021-11-08	2021-08-16	2021-09-20	16
NT4	Northwest Territories	2021-08-16 to 2021-11-01	2021-08-19	2021-08-30	12
NT5	Northwest Territories	2021-06-30 to 2021-11-23	2021-08-12	2021-09-23	53
NT6	Northwest Territories	2021-08-19 to 2021-11-16	2021-08-19	2021-08-26	23
NU1	Nunavut	2021-07-14 to 2021-11-29	2021-09-10	2021-11-15	54
NU2	Nunavut	2021-07-22 to 2021-11-29	2021-08-17	2021-09-08	56
ON1	Toronto, ON	2021-07-11 to 2021-11-30	2021-07-11	2021-08-29	38
ON2	Toronto, ON	2021-07-11 to 2021-11-28	2021-07-18	2021-07-27	37
ON3	Toronto, ON	2021-07-11 to 2021-11-30	2021-07-18	2021-08-15	37
ON4	Toronto, ON	2021-07-11 to 2021-11-30	2021-08-03	2021-09-14	38
QC1	Montréal, QC	2021-07-14 to 2021-12-01	2021-07-21	2021-09-08	36
QC2	Montréal, QC	2021-07-17 to 2021-12-01	2021-08-11	2021-10-16	36

Abbreviations: AB, Alberta; BC, British Columbia; NL, Newfoundland and Labrador; NS, Nova Scotia; NT, Northwest Territories; NU, Nunavut; ON, Ontario; QC, Québec
